# Endobronchial Leiomyoma Presenting as Post‐Obstructive Pneumonia

**DOI:** 10.1002/rcr2.70124

**Published:** 2025-02-18

**Authors:** Boon Hau Ng, Hsueh Jing Low, Nik Nuratiqah Nik Abeed, Nor Safiqah Sharil, Rose Azzlinda Osman, Maalini Krishnasamy, Andrea Yu‐Lin Ban, Jamalul Azizi Abdul Rahaman

**Affiliations:** ^1^ Respiratory Unit, Department of Medicine, Hospital Canselor Tuanku Muhriz, Faculty of Medicine Universiti Kebangsaan Malaysia Kuala Lumpur Malaysia; ^2^ Department of Anesthesiology and Critical Care, Hospital Canselor Tuanku Muhriz, Faculty of Medicine Universiti Kebangsaan Malaysia Kuala Lumpur Malaysia; ^3^ Department of Medicine Thomson Hospital, Kota Damansara Petaling Jaya Petaling Jaya Malaysia

**Keywords:** cryo‐resection, leiomyoma, pneumonia, rigid bronchoscopy

## Abstract

Endobronchial leiomyoma is a rare airway tumour. We report the case of a 30‐year‐old female non‐smoker who presented with persistent fever and cough, ultimately diagnosed with post‐obstructive pneumonia secondary to an endobronchial leiomyoma. The patient underwent a successful endobronchial cryo‐resection with the resolution of symptoms. This case highlights the importance of considering rare causes of non‐resolving pneumonia and the role of a minimally invasive approach in the management of endobronchial leiomyoma.

## Graphical abstract

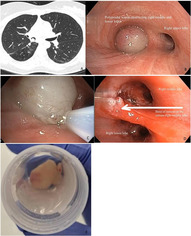

## 1 Introduction

Benign central airway tumours are uncommon and generally comprise hamartomas and papillomas. Tracheobronchial leiomyomas are rare, representing only about 0.6% of all benign lung neoplasms [1]. They can present as persistent pneumonia due to bronchial obstruction, which may mimic more common infectious or neoplastic conditions. Here, we describe a case of endobronchial leiomyoma presenting with post‐obstructive pneumonia in a young female who was successfully treated with rigid bronchoscopy‐guided endobronchial cryo‐resection.

## 2 Case Report

A 30‐year‐old female non‐smoker presented with a 10‐day history of cough and fever. Her medical history was unremarkable. On examination, her vital signs revealed a blood pressure of 106/62 mmHg, a heart rate of 110 beats/min, a temperature of 38°C, and SpO₂ of 93% on room air. Lung auscultation revealed reduced breath sounds over the right lower zone.

Laboratory investigations showed leukocytosis with a total white cell count of 18 × 10^9^/L and elevated C‐reactive protein (CRP) at 22 mg/L. Arterial blood gas analysis on room air revealed a pH of 7.42, pO₂ of 70 mmHg, pCO₂ of 40 mmHg, and bicarbonate of 24 mmol/L. A chest radiograph showed consolidation in the right middle and lower zones. The patient was diagnosed with community‐acquired pneumonia and started on intravenous amoxicillin–clavulanate and azithromycin.

Despite 72 h of antibiotic therapy, the fever persisted, and a repeat chest radiograph showed no resolution of the right middle and lower zone consolidation. Serum procalcitonin was elevated at 8 ng/mL, prompting escalation to intravenous piperacillin‐tazobactam. Sputum cultures, acid‐fast bacilli (AFB) staining, *Mycobacterium tuberculosis* GeneXpert, and cytological analysis were negative.

**FIGURE 1:**
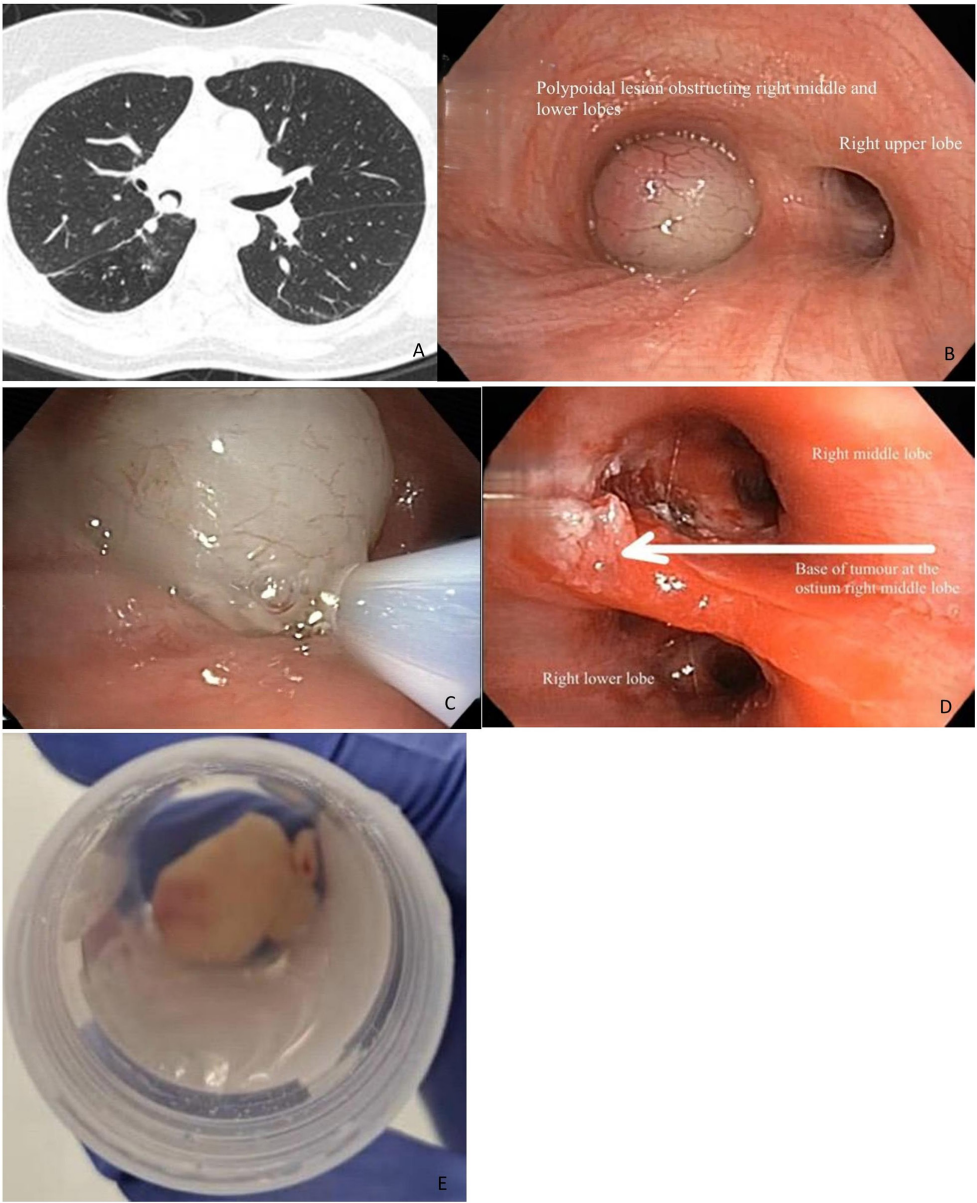
(A) CT showed right bronchus intermedius soft tissue density. (B) Bronchoscopic view of the polypoidal mass within the right bronchus intermedius. (C) Picture showing cryo‐resection. (D) Post‐cryo‐resection showing recanalisation of the right middle and right lower lobes bronchus. (E) Gross appearance of the mass after endobronchial electrocautery snare and cryoextraction.

Contrast‐enhanced computed tomography (CECT) of the thorax revealed a collapsed right lower lobe with the right lower lobe bronchus occluded by an intraluminal soft‐tissue mass (Figure 1A). Flexible bronchoscopy identified a smooth, lobulated mass occluding the right bronchus intermedius (Figure 1B), preventing further passage of the bronchoscope due to total occlusion of the right bronchus intermedius. Bronchoalveolar lavage was negative for bacterial and mycobacterial pathogens. A diagnosis of post‐obstructive pneumonia secondary to an endobronchial mass was made.

Following a multidisciplinary team discussion, the patient underwent rigid bronchoscopy with endobronchial cryo‐resection under general anaesthesia. An EFER‐Dumon bronchial tube 11.00–12.00 was used for intubation. An electrocautery snare was used to resect the mass. A cryoprobe was used to freeze and remove the mass via the rigid bronchoscope (Figure 1C). However, the mass was too large for the lumen of the rigid bronchoscope, so the cryoprobe and the rigid bronchoscope were removed from the airway en bloc. The patient was re‐intubated with the rigid bronchoscope, and the remaining mass was resected using an electrocautery snare. Purulent secretions were seen from the right middle lobe and right lower lobe. The right bronchus intermedius was successfully re‐canalised, and the base of the mass was seen in the ostium of the right middle lobe (Figure 1D,E). The patient had an uneventful postoperative course. Post‐obstructive pneumonia resolved completely after the mass removal.

**FIGURE 2:**
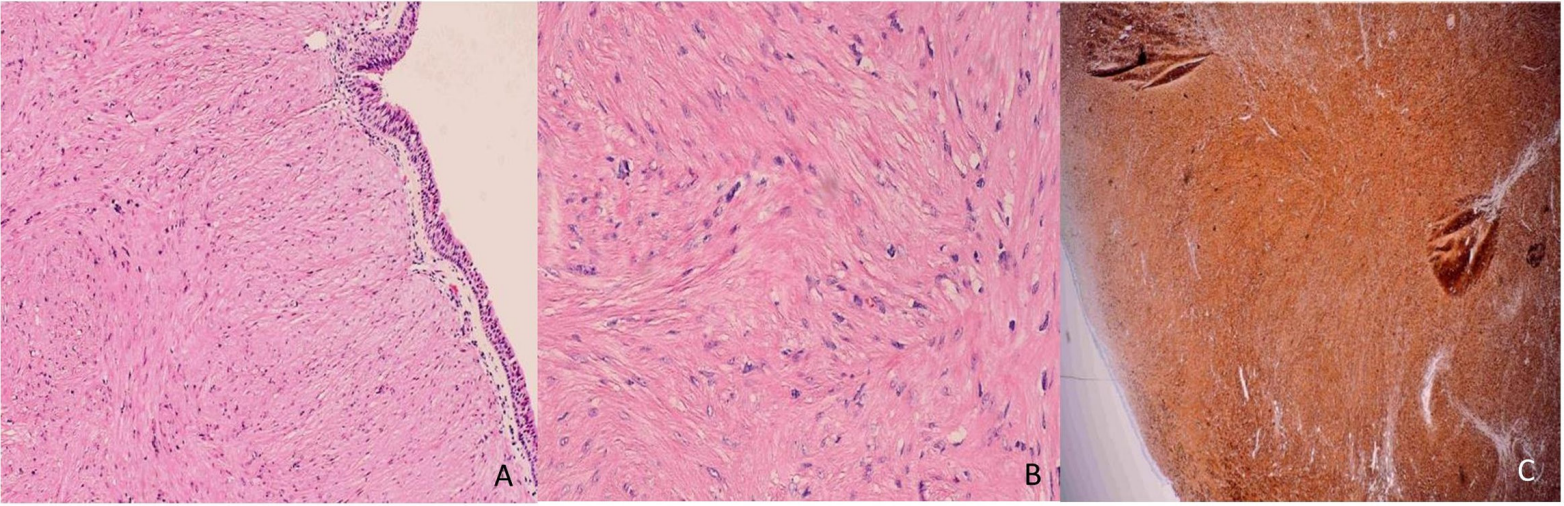
(A) A low‐power view showing the lesion covered by benign bronchial epithelium (haematoxylin and eosin [H&E] staining, ×100). (B) A medium power view demonstrating smooth muscle cells without evidence of atypia or malignancy. There is no cytological atypia, necrosis, increased or atypical mitotic activity (haematoxylin and eosin [H&E] staining, ×200). (C) The spindle cells are strong and diffusely positive for H‐caldesmon, a specific marker for smooth muscle differentiation (IHC staining, ×200).

Histopathological examination revealed a circumscribed lesion covered by bronchial epithelium, composed of interlacing fascicles of spindle cells consistent with smooth muscle origin. The spindle cells exhibited elongated nuclei with fine chromatin and eosinophilic cytoplasm. Immunohistochemistry showed positivity for h‐caldesmon and negativity for CD117 and S100, with a low Ki‐67 proliferation index of 10% (Figure 2A–C). The lesion tested negative for oestrogen receptor (ER) and progesterone receptor (PR). These findings confirmed the diagnosis of endobronchial leiomyoma.

## 3 Discussion

Primary bronchial neoplasms, whether benign or malignant, are rare. The majority of primary bronchial tumours are malignant, with adenoid cystic carcinoma and squamous cell carcinoma being the most common histological types [2]. Among benign bronchial tumours, pleomorphic adenoma is the most frequently encountered, whereas endobronchial leiomyoma is one of the rarest, accounting for approximately 2% of benign lung tumours [3]. Pulmonary leiomyomas can occur in various locations, including intraparenchymal, endotracheal, or endobronchial sites. About one‐third of these tumours are present in endobronchial locations [4]. These tumours predominantly affect individuals in their fourth decade of life and have a female predilection.

The clinical presentation of bronchial leiomyoma varies depending on the tumour's location, size, and degree of bronchial obstruction. Parenchymal or peripheral pulmonary leiomyomas often manifest as asymptomatic solitary nodules. In contrast, endobronchial leiomyomas typically present with symptoms such as cough, wheezing, haemoptysis, and recurrent episodes of pneumonitis secondary to partial or complete bronchial obstruction.

Fiberoptic bronchoscopy is the preferred diagnostic modality to evaluate the tumour's location, appearance, and extent. It also facilitates tissue sampling for histological confirmation. A definitive histological diagnosis is critical for determining the appropriate surgical intervention, enabling limited resection rather than more radical procedures. However, bronchoscopy alone has limitations, particularly in assessing extraluminal involvement and distal airway patency.

Computed tomography (CT) is a complementary imaging tool that provides detailed visualisation of bronchial leiomyomas. On unenhanced CT, these tumours typically have an attenuation of 25–46 Hounsfield units, increasing to 46–85 Hounsfield units on contrast‐enhanced scans [5]. When confined to the bronchial lumen, leiomyomas appear as homogeneous, smooth or lobulated masses with well‐defined margins and diffuse enhancement [6]. Virtual bronchoscopy and three‐dimensional reconstruction of high‐resolution CT scans further enhance diagnostic accuracy by providing multidirectional visualisation of intraluminal lesions.

Histologically, bronchial leiomyomas are composed of disorganised smooth muscle bundles with minimal vascular or fibrous components. Immunohistochemical staining typically demonstrates strong positivity for smooth muscle markers, including smooth muscle actin (SMA), desmin, and smooth muscle myosin heavy chain [7]. The absence of mitotic activity, necrosis, or cellular atypia is essential to rule out leiomyosarcoma.

The prognosis of bronchial leiomyomas is excellent following complete resection. However, recurrences may occur, particularly when the tumour exhibits an iceberg growth pattern with both intraluminal and extraluminal components [5, 8]. In such cases, bronchoscopic removal of only the visible intraluminal tumour may lead to local recurrence [9].

The modality of treatment depends on the tumour's size, location, and extent. Traditionally, lobectomy or pneumonectomy has been the standard approach for resecting endobronchial leiomyomas [10]. Less invasive techniques, such as bronchoscopic removal, Nd:YAG laser ablation, electrocautery, snare excision, bronchoplasty, and bronchotomy with sleeve resection, have been reported as safe alternatives [8, 10, 11]. However, these methods are limited by anatomical and technical challenges. Parenchymal resection remains appropriate for solitary parenchymal nodules.

In the present case, we opted for rigid bronchoscopy with cryoresection, which provided a less invasive yet effective alternative for tumour removal. However, due to the risk of recurrence, endoluminal resection methods often require multiple sessions and lifelong surveillance [12].

Bronchial leiomyoma is an uncommon cause of bronchial obstruction. While traditional surgical approaches remain effective, rigid bronchoscopy with cryo‐resection offers a minimally invasive and safe alternative for managing these rare tumours.

## Author Contributions

B.H.N., H.J.L., N.N.N.A., and N.S.S. wrote up the case under the continuous supervision of A.B.Y.L. and J.A.A.R. They discussed the case presentation, investigations, and patient management. R.A.O. and M.K. were involved in the patient's management. J.A.A.R. and A.B.Y.L. supervised and guided the patient's management.

## Ethics Statement

The authors declare that appropriate written informed consent was obtained for the publication of this manuscript and accompanying images.

## Conflicts of Interest

Andrea Ban Yu‐Lin is an Editorial Board member of Respirology Case Reports and a co‐author of this article. She was excluded from all editorial decision‐making related to the acceptance of this article for publication.

## Data Availability

The data that support the findings of this study are available from the corresponding author upon reasonable request.
